# Hippocampus and its involvement in Alzheimer’s disease: a review

**DOI:** 10.1007/s13205-022-03123-4

**Published:** 2022-02-01

**Authors:** Y. Lakshmisha Rao, B. Ganaraja, B. V. Murlimanju, Teresa Joy, Ashwin Krishnamurthy, Amit Agrawal

**Affiliations:** 1 Department of Anatomy, Kasturba Medical College, Mangalore, Manipal Academy of Higher Education, Manipal, Karnataka India; 2 Department of Physiology, Kasturba Medical College, Mangalore, Manipal Academy of Higher Education, Manipal, Karnataka India; 3 Department of Anatomy, College of Medicine, American University of Antigua, Coolidge, Antigua, Antigua and Barbuda; 4 Department of Anatomy, K.S. Hegde Medical Academy, Deralakatte, Nitte University, Mangalore, Karnataka India; 5 Department of Neurosurgery, All India Institute of Medical Sciences, Saket Nagar, Bhopal, 462020 Madhya Pradesh India

**Keywords:** Alzheimer’s disease, Hippocampus, Sirtuin 1, Tau proteins

## Abstract

Hippocampus is the significant component of the limbic lobe, which is further subdivided into the dentate gyrus and parts of Cornu Ammonis. It is the crucial region for learning and memory; its sub-regions aid in the generation of episodic memory. However, the hippocampus is one of the brain areas affected by Alzheimer’s (AD). In the early stages of AD, the hippocampus shows rapid loss of its tissue, which is associated with the functional disconnection with other parts of the brain. In the progression of AD, atrophy of medial temporal and hippocampal regions are the structural markers in magnetic resonance imaging (MRI). Lack of sirtuin (SIRT) expression in the hippocampal neurons will impair cognitive function, including recent memory and spatial learning. Proliferation, differentiation, and migrations are the steps involved in adult neurogenesis. The microglia in the hippocampal region are more immunologically active than the other regions of the brain. Intrinsic factors like hormones, glia, and vascular nourishment are instrumental in the neural stem cell (NSC) functions by maintaining the brain’s microenvironment. Along with the intrinsic factors, many extrinsic factors like dietary intake and physical activity may also influence the NSCs. Hence, pro-neurogenic lifestyle could delay neurodegeneration.

## Introduction

The cerebral cortex and hippocampus are closely associated with cognitive function and neurogenesis in the brain (Hu et al. 2019). Along with the β-amyloid and tau proteins, trans-active response deoxyribonucleic acid (TAR DNA)-binding protein of 43 kDa (TDP-43) is also a newly linked protein in the AD. TDP-43 protein accumulates in the amygdala and spreads into the hippocampus, leading to faster atrophy (Josephs et al. 2017). Hence, prevention of spreading this protein into the hippocampus could treat AD (Josephs et al. 2017). The genetic neuroimaging works have proved that the AD risk alleles show a crucial role in cortical and hippocampal morphometry (Lancaster et al. 2019). In AD, neuronal degeneration happens because of the accumulation of two abnormal proteins, β-amyloid, and tau, in the brain. The characteristic feature of AD is an excessive formation or decreased elimination of amyloid-beta (Aβ). Microscopically and pathologically, AD is described as the extracellular accumulation of senile plaques in addition to the intracellular accumulation of the neurofibrillary tangles in various parts of the brain, particularly in the hippocampus (Chu 2012).

Along with the deposition of abnormal proteins, the AD brain also shows the degeneration of the hippocampus (Josephs et al. 2017). Hippocampus is a vital region of the memory, and this part of the brain shows adult neurogenesis (Poo et al. 2016). In old age, the hippocampus is affected by cognitive decline, which is also seen in neurodegenerative disorders like AD (Jaroudi et al. 2017). Though hippocampal degeneration is also seen in other neurodegenerative diseases like Lewi body dementia, the degree of deterioration is markedly more in AD. The knowledge of variation in the hippocampus will help diagnose and treat AD (Elder et al. 2017). The patients with AD are clinically presented with the deterioration of activity of daily living due to the cognitive and functional decline (Chu 2012).

### Subdivisions of hippocampus (Fig. 1) and the Papez circuit

**Fig. 1:**
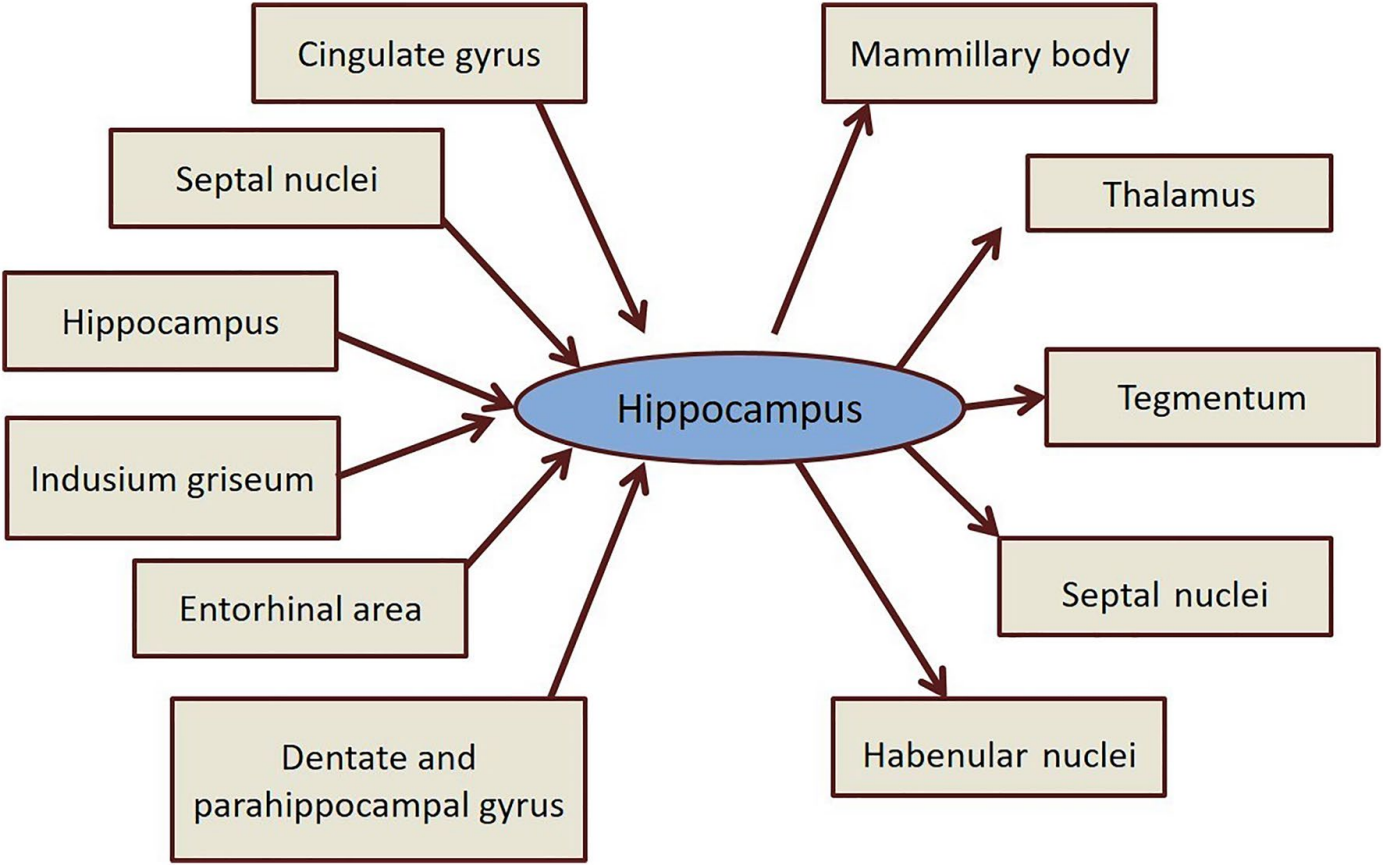
Afferent and efferent connections of hippocampus

The subdivisions of the hippocampus include the dentate gyrus (DG) and components of cornu ammonis (CA1, CA2, CA3, and CA4 regions). The DG contains tightly arranged small granule cells, which cover the terminal part of the hippocampus near the hippocampal fissure (Mufson et al. 2015), and the CA regions contain densely packed pyramidal cells (Hyman et al. 1987). The CA4 region emerges from the DG and continues as CA3, CA2, and a narrow strip, CA1. The narrow strip of CA1 merges with the subiculum. Papez in 1937 described a circuit, which connects the cingulate gyrus to the hippocampus through the mammillary bodies and anterior thalamus. This circuit is popularly known as the Papez circuit. According to Papez, sensory inputs of emotion are transported to the hippocampus via the cingulate gyrus. The emotional expressions are organized in the hippocampus and expressed via the mammillary bodies. The hypothalamus helps in the peripheral terms of the emotional state by providing access to the autonomic outflow into the hippocampal processes. The Papez circuit is extensively involved in the mnemonic functions, spatial short-term memory, and cognitive processes (Brennan et al. 2019).

### Microanatomy of hippocampus

The hippocampus is a trilaminate archicortex, which is made up of upper and lower plexiform layers, and in between these, there is a pyramidal cell layer. The CA1, CA2, CA3, and CA4 are the four fields of the hippocampus (Brennan et al. 2019). The CA3 region receives mossy fibers from the DG, and it contains about ten layers of pyramidal cells; these cells are the largest among the hippocampal pyramidal cells. The CA2 region has compactly arranged pyramidal cells, which receive significant inputs from the supra-mammillary areas of the hypothalamus. The CA1 region overlaps the subiculum, and about 10% of the neurons present in the CA1 region are interneurons. The three layers of the hippocampus are included in the six strata. These strata are named as the alveus, stratum orience, stratum pyramidalis, stratum lucidum, stratum radiatum, and stratum lacunosum-moleculare (Brennan et al. 2019). In the sub-granular layer of DG, the proliferation of neurons occurs throughout the life span. This neurogenesis is essential in the hippocampal function, like learning, long-term memory, spatial memory, and mood.

### Blood supply of hippocampus

The hippocampus is usually supplied by the branches of the posterior cerebral artery, a branch of the basilar artery, and the anterior choroidal artery, an extension of the internal carotid artery. However, the central part of the hippocampus is supplied by the posterior cerebral artery. It is also reported that the arterial supply of the hippocampus may vary in some people (Spallazzi et al. 2019). The arteries of the hippocampus are named according to their area of distribution. The anterior hippocampal artery supplies the head and uncus; the body is provided by the middle hippocampal artery, whereas the posterior hippocampal artery supplies the tail of the hippocampus. The arteries supplying the hippocampus are situated outside the ventricular cavity. On the subiculum region, which is located at the dorsal part of the para-hippocampal gyrus, the hippocampal arteries run parallel to the hippocampus and give rise to straight arteries. All these arteries enter the hippocampus at the level of DG. The anatomy of vascularisation of the hippocampus is vital to the neurosurgeons during the surgeries of this region of the brain.

### Connections of hippocampus (Fig. 2)

**Fig. 2:**
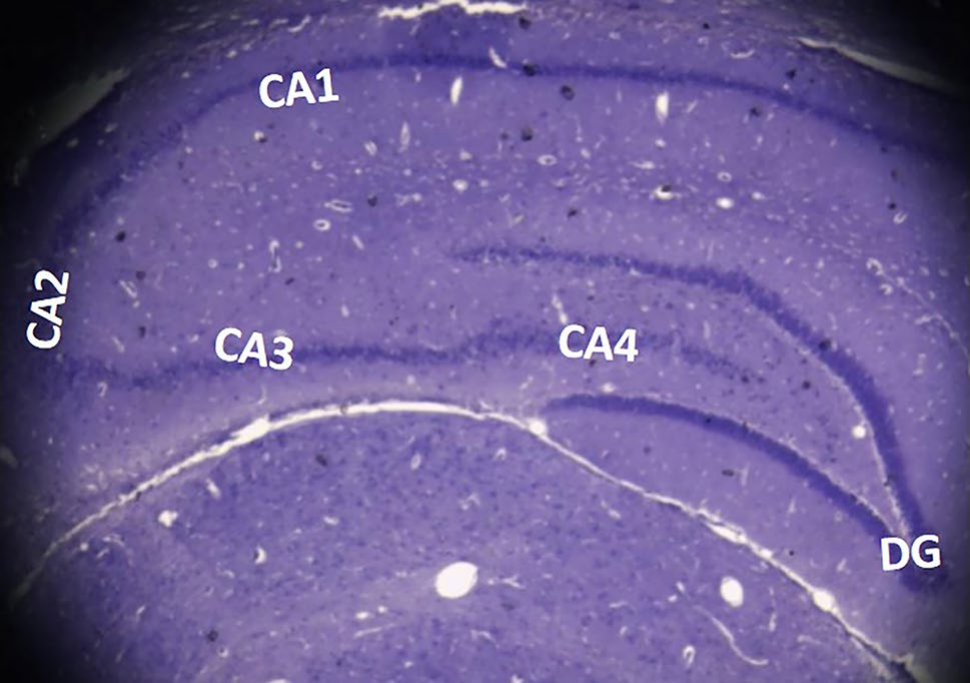
Cresyl violet staining in an *Albino Wistar* rat model showing (2x) the subdivisions of hippocampus (DG-dentate gyrus; CA1-cornu ammonis region 1; CA2-cornu ammonis region 2; CA3-cornu ammonis region 3; CA4-cornu ammonis region 4)

The significant afferent connections to the hippocampus come from the entorhinal cortex via the perforant path and via the fimbria/fornix from the basal forebrain and brain stem. Supra-mammillary nucleus (SUM) and the reuniens nucleus (RE) influence the learning process and memory (Vertes 2015). The hippocampus is responsible for episodic memory. The CA2 and CA3 regions of the hippocampus will receive input from all the sources, and this information will be segregated (Lisman et al. 2017). The association fibers from the rostrocaudal levels of the hippocampus and subcortical structures like septal nuclei and supra-mammillary regions reach the stratum radiatum and stratum orience of CA3 and CA2 regions. The projection fibers from the CA3, CA2, and CA1 areas will also reach the stratum radiatum and stratum orience. These projection fibers are called Schaffer’s collaterals. The projection fibers from the entorhinal cortex travel through the stratum lacunosum-moleculare, making synapsis with the dendrites of the pyramidal cells of the hippocampus. This pathway is called the perforant pathway. It was reported that the disruption of the perforant path would lead to defects in learning and memory (Ginsberg 2010).

### Hippocampus and memory

The hippocampal sub-regions aid in the process of the generation of episodic memory (Langnes et al. 2020; Collin et al. 2015). The hippocampal-dependent memory signals outline memory formation, an active and ongoing process in the brain (Voss et al. 2017). According to de Landeta et al. (2020), the retrosplenial cortex in the occipital lobe plays a role in the long-term object recognition memory, similar to the hippocampus. The CA3 region of the hippocampus produces the sharp-wave ripples (SWR), which propagate the recent memory traces into the neocortex for consolidation of the memory (Karimi Abadchi et al. 2020). Supporting de Landeta et al. (2020) opinion, Karimi Abadchi et al. (2020) also found that the maximum SWR modulation occurs in the retrosplenial cortex. The excitatory output of SWR affects a larger area of the cortex along with the subcortical nuclei. This activity occurs during the ‘off-line’ state of the brain (Buzsáki 2015). The hippocampus proper is responsible for episodic memory (Knierim 2015). Samuel et al. (1994) observed that the neurofibrillary tangle (NFT) accumulation in the hippocampal region is related to dementia. The NFT accumulation in the stratum lacunosum, dentate fasciculus, CA2, CA3, and CA4 areas had resulted in the synaptic loss. During the early learning stage, the neocortical prefrontal engram cells are generated by the input from the hippocampus, entorhinal cortex, and basolateral amygdala. These prefrontal engram cells, with the aid of engram cells of the hippocampus, will eventually mature. Hence, the neocortical engram cells are a critical part of remote recollection (Kitamura et al. 2017; Roy et al. 2016). The oxidative damage can reduce the cognitive function in the cortex and hippocampus (Fonzo et al. 2009). The DG is one of the brain regions which shows neurogenesis throughout the life span of a mammalian. The DG, being an information gateway, has a significant contribution to the formation of episodic memory of the hippocampus (Poo et al. 2016; Eriksson et al. 1998; Altman and Das 1965).

### Adult neurogenesis in hippocampus

Proliferation, differentiation, and migrations are the steps involved in adult neurogenesis. The sub-granular zone of DG and the sub-ventricular zone are the two regions consisting of neural stem cells (NSCs) of the adult brain, which show neurogenesis (Abbott and Nigussie 2020). The B1 cell residues, which line the junction between the stratum and lateral ventricle, possess the astroglial property and these cell residues act as the NSCs (Horgusluoglu et al. 2017). These NSCs, responsible for adult neurogenesis, are retained in the sub-ventricular and sub-granular zone regions of the DG in the adult brain. The olfactory bulb and DG are the two regions where neurogenesis is seen throughout life (Horgusluoglu et al. 2017). In the brain, neurogenesis occurs in a niche, where the NSCs are located near the blood vessels. Therefore, signals from the existing neural cells and nearby vasculature will stimulate the NSCs for neurogenesis (Ozek et al. 2018; Shen et al. 2008). Impairment in adult neurogenesis may be a critical factor for neurodegenerative disorders like AD and Parkinson’s disease. Genetic mutation, brain injury, and aging may cause depletion in the function of neural precursors (Shohayeb et al. 2018). The intrinsic factors like hormones, glia, and vascular nourishment will play a leading part in the role of NSCs by maintaining the microenvironment of the brain (Shohayeb et al. 2018; Licht and Keshet 2015).

Besides these intrinsic factors, few extrinsic factors like dietary intake and physical activity may also influence the NSCs. Hence it is understood that pro-neurogenic lifestyle could delay neurodegeneration. Neurotrophic factors (NTFs) can help the diseased neurons in AD and are offered through the viral vectors. The viral vectors can be inserted directly over that particular region, which has neuronal degeneration, and it will lead to the transduction of cells, which secrete the NTF. Besides this, stem cells may also help in neurodegenerative disorders like AD and Parkinson’s disease (Shohayeb et al. 2018).

### Hippocampal lesions in AD

The neuropathological abnormality in AD includes neuronal loss and gliosis at the hippocampus (Ball et al. 1985). The volumes of hippocampal layers like stratum radiatum, stratum lacunosum, stratum moleculare and subiculum’s, stratum pyramidale are bilaterally lost in the patients of AD (Boutet et al. 2014). Though the etiology of AD is not clearly understood, the pathophysiology of AD demonstrates the neuro-inflammation, accumulation of Aβ peptides, phosphorylated tau, and oxidative stress (Reddy et al. 2017, 2012). The entorhinal area is the first region, where the plaques and tangles are deposited in AD patients (Knierim 2015). In the early stage of AD, tau protein gets accumulated in the entorhinal cortex and later spreads into the hippocampus (Asai et al. 2020). The anatomical and histological studies of autopsied AD brains have revealed that the neurodegeneration starts in the second layer of the entorhinal cortex and gradually extends into the hippocampus, temporal cortex, frontoparietal cortex and subcortical nuclei. In the later stages of the disease, there will be a disconnection between the DG and sub-regions of the hippocampus, leading to cognitive disorders (Reddy et al. 2018; Samuel et al. 1994; Hyman et al. 1986). Du et al. (2016) observed the accumulation of tau-1 positive foci in the polymorphic layer of the DG of rat models 7 days after the blast-induced traumatic cerebral injury. In AD, NFTs are first accumulated in the CA1 area of the hippocampus, then gradually affect the subiculum, CA2, CA3, and DG (de Flores et al. 2015; Lace et al. 2009). The tau positive neurons are associated with the Aβ deposits through their axonal projections in the AD brain. It was reported that Aβ deposition reduces the inputs in the hippocampus (Lace et al. 2009).

### Hippocampal microglia in AD

Microglia are the resident cells of the brain, which play an essential role in regulating developmental processes like neurogenesis, axonal growth, myelination, synaptic maturation, and synaptic pruning. Microglia show significant changes during hippocampal development from the 14th postnatal day to the 28th postnatal day (Delpech et al. 2016; Parkhurst et al. 2013). The structural and functional deficiencies of genes like CX3 chemokine receptor 1 (CX3CR1), colony-stimulating factor 1 (CSF-1), methyl-CpG binding protein 2 (Mecp -2), and Homeobox B8 (Hox B8) can affect the microglia, which has an impact on the normal functioning of the central nervous system (Dantzer et al. 2008). It was reported that microglia in the hippocampal region are more immunologically active than the other regions of the brain (van Olst et al. 2020; Grabert et al. 2016). The microglial genes play an essential role in refining the neural circuits by removing the redundant synaptic connections (Rajendran and Paolicelli 2018). As innate immune effector cells, the microglia are vital in the amyloid-beta clearance (Rivera-Escalera et al. 2019). Microglial activation is a usual phenomenon during the onset of AD and is associated with elevated inflammatory markers in the cerebrospinal fluid (Sarlus and Heneka 2017). In the hippocampus of the AD brain, the elevation of pro-inflammatory cytokines like interleukin one β (IL-1β) will induce the microglial proliferation, which will further help remove the fibrillar Aβ (Rivera-Escalera et al. 2019). Bachstetter et al. (2015) described that the mutations in CD33 and triggering receptors expressed on myeloid cell 2 (TREM2) genes could lead to AD. In the progression of AD, atrophy of medial temporal and hippocampal regions are the structural markers in the MRI (Grabert et al. 2016). The T1- weighted scans are most commonly used, as they provide good contrast between the white and grey matter. Jacket al. (1999) observed an association between the premorbid hippocampal volume and crossing over to AD.

Microglia activation is associated with the etiopathogenesis of numerous degenerative diseases in the central nervous system (Navarro et al. 2018). It was reported that the microglial degeneration, which is observed in the DG of AD patients, is due to the toxic soluble phospho-tau species accumulation (Navarro et al. 2018). However, the role of microglia in AD has not been clarified (Sanchez-Mejias et al. 2016). It was described that compromised activities of the microglia and its altered response to the β-amyloid could increase the risk of AD (Hansen et al. 2018). The microglia are neuroprotective, and they act as phagocytes to engulf the Aβ, thereby preventing AD. The microglia are activated into disease-associated microglia (DAM) state with apolipoprotein E (apoE), which depends on the TREM2. Unfortunately, because of genetic susceptibility and aging, the function of microglia will become insufficient to prevent AD development. There will be an accumulation of toxic amyloid species, leading to tauopathy in the stressed and damaged neurons (Hansen et al. 2018). The microglia will be changed into an inflammatory state, leading to the engulfing of synapses and secretion of neurotoxic cytokines, eventually injuring the neurons. In the later stages of AD, there will be synaptic loss and symptomatic decline. It was reported that microglia have dual faces, one of them is favorable, and the other is detrimental (Rao et al. 2020). This dual nature of microglial function will complicate the management protocol of AD since AD targets the microglia. The stimulation of microglia is helpful in the initial stages of AD, which eventually becomes unfavorable during the neurodegenerative stage (Hansen et al. 2018).

### Growth factors and the hippocampus

The nerve growth factor (NGF), neurotrophin 3 (NT-3), neurotrophin 4/5 (NT-4/5), and the brain-derived neurotrophic factor (BDNF) are the proteins, which specifically help the survival of neurons in AD patients (Hock et al. 2000). BDNF is one of the significant neurotrophins, which protect the neurons by promoting their survival against the various neuronal insults in different brain regions, including the hippocampus (Rai et al. 2013). The primary source for the BDNF is the neuron. Apart from the neurons, astrocytes and microglia also contain the BDNF (Lisman et al. 2017). The oxidative stress can reduce the BDNF expression, thereby leading to hippocampal atrophy. Therefore, antioxidants can reduce oxidative damage, which occurs due to the oxidative stress. It was reported that levels of ratios of NT-4/NT-3, NT-4/NT-5, and BDNF had been considerably declined at the hippocampus of AD models (Hock et al. 2000). However, the intensities of NGF/NT-3 ratio and NGF were increased at the hippocampus of AD animal models. Hocket al. (2000) suggested that declined intensities of the BDNF might lead to neuronal degeneration in AD. It was reported that NGF and BDNF had protected the basal forebrain cholinergic neurons from the degeneration, which was induced by the axotomy (Knusel et al. 1992). Along with this, neurons containing 5-hydroxytryptamine are also decreased in the brain, which causes depression and behavioral changes in AD (Raskind and Peskind 1994).

### Growth differentiation factor (GDF) and hippocampal vasculature

The neurogenesis occurs at the niche, where the neural stem cells (NSCs) are seen near the blood vessels. The signals from the neurons and blood vessels will influence the NSC proliferation and differentiation. Several animal experiments have shown that the systemic factors in the circulation positively influence brain neurogenesis. Ozek et al. (2018) observed that the administration of GDF 11 has improved the hippocampal vascular niche in old mice. They have also reported that systemic administration of GDF 11 has elevated the newborn neuron (Bromodeoxyuridine, BrdU^+^/ neuronal nuclear protein, NeuN^+^), neural stem cells (sex-determining region, Sox2^+^), and neural progenitor/immature neurons (doublecortin, DCX^+^). Ozek et al. (2018) observed that GDF 11 had improved the vasculature of the hippocampus by increasing the blood vessels and their branches. But these improvements were honored to be only seen in the old mice brains.

It was reported that, genetic activation of activing—like kinase 5 (ALK 5) receptor for the GDF 11, has improved the neurogenesis in hippocampus by activating the downstream signalling through spinal muscular atrophy (Sma-) and mothers against decapentaplegic (Mad-)-related proteins 2/3 (SMAD 2/3). Hence, GDF11 improves hippocampal neurogenesis and vasculature (Ozek et al. 2018). The GDF-11 is present in the bloodstream, with some of it crossing the blood–brain barrier (Ozek et al. 2018). It is believed to exhibit several beneficial effects on the vasculature by promoting angiogenesis, maintenance of blood–brain barrier and vascular stability (Sutherland et al. 2020). Aging will cause impaired neurogenesis due to the decreased vascular density and blood flow. The brain of elderly individuals is predisposed to many stressors, leading to irreversible brain damage. The recent trends suggest that GDF-11, which is present in young people's blood, can have neuroprotective effects (Lu et al. 2018). It was reported that the circulating GDF-11 in the blood decreases with the increasing age (Poggioli et al. 2016). This suggests that patients with AD may have reduced levels of GDF in their blood.

### Function of NSCs in hippocampus of AD patients

NSCs have the self-renewal capacity and can differentiate into cells like neurons, oligodendrocytes, and astrocytes. The differentiated neurons help organize complex neuronal circuits, which are essential during the processing of information and transmission (Kino 2015). NSCs, after becoming the neuroglial cells like astrocytes and oligodendrocytes, will help in the proper functioning of neurons. DG of the hippocampus contains plenty of these NSCs (Androutsellis-Theotokis et al. 2008), which help in learning and memory. NSCs also increase the response to anti-depressant drugs and help in the subsequent recovery (Kino 2015). The adult NSCs in the hippocampus can grow and maintain their number throughout life (Zhang et al. 2015). NSCs are involved in consolidating, preserving, and organizing the memory, which is mediated by the hippocampus. They also assist in mood and behavioral integrity. NSCs are involved in the turnover of pyramidal cells, which is important for replacing old memory traces with new ones. NSCs are described to have their capacity in neuronal regeneration. The NSC transplantation therapy is a favourable means of managing neuronal diseases and restoration of microenvironment at the injury site (Tang et al. 2017). They secrete soluble factors, which include cytokines, growth factors, and neurotrophic factors. These factors can protect the existing neurons against the damage in situ. AD is caused by neuronal or neuroglial cell defects, resulting in memory deterioration, cognitive disorders, dementia, and body movement disorders.

It was described that hippocampal neuronal mitochondria are decreased in AD patients. The transplantation of NSCs into the transgenic AD mice model has shown a significant increase in the mitochondrial number and expression of mitochondria-related proteins. This has improved the cognitive function in a mouse model (Zhang et al. 2020). Human NSCs have been tried to treat AD in transgenic animal models (Li et al. 2016; Blurton-Jones et al. 2014). It was reported that intranasal administration of NSCs showed the ability to migrate to different regions of the central nervous system, including the hippocampus, cerebral cortex and spinal cord (Zhang et al. 2020). Though most of the parts of the brain are affected in AD, the regions which are essential for the learning and memory like hippocampus, entorhinal cortex, and basal forebrain show marked degeneration. Decreased neurotrophin expression and its impaired neuronal transport are apparent in the neurodegenerative brain. Hence, maintaining the regular neurotrophin expression can be a treatment modality in AD management. Marsh and Blurton-Jones (2017) have demonstrated the potential benefits of NSCs in providing neurotrophic support in AD.

### Sirtuins and the hippocampus

Sirtuins (SIRT), belonging to the family of histone deacetylases, are known to have a significant role in maintaining genomic stability. SIRT are classified into seven types, which are numbered from one to seven. SIRT1 is involved in neurological conditions like AD and Parkinson's disease. The pathogenic mechanism in these disorders has been linked to apoE. It is observed that the expression of apoE4 reduces the SIRT1 level. Hence, it is considered a genetic risk factor in AD (Cacabelos et al. 2019; Jęśko et al. 2017). SIRT1, a nicotinamide adenine dinucleotide + (NAD+)-dependent deacetylase, acts as an intracellular regulatory protein and helps in the cell survival. SIRT1 can also preserve the mitochondrial function in the neuron and modulate the responses to DNA damage. Recent studies have reported that SIRT1 activity is increased in the rat hippocampus following the status epilepticus. Hence, activating the SIRT1 pathway may enhance the mitochondrial biogenesis and can act as an endogenous neuroprotective agent (Chuang et al. 2019).

Decreased SIRT level, particularly SIRT1, is associated with Aβ deposition. Decreased levels of SIRT1 may result in the accumulation of Aβ peptides (Koo et al. 2017; Julien et al. 2009). Lack of SIRT1 expression in hippocampal neurons will impair cognitive function, including recent memory, spatial learning (Kumar et al. 2017). Hence, SIRT1 regulates neuronal survival, insulin sensitivity, glucose metabolism, and mitochondrial biogenesis and thus maintains neuronal homeostasis (Gomes et al. 2018; Satoh and Imai 2014; Guarente 2013). Resveratrol, an antioxidant used to manage AD, upregulates the SIRT1 and decreases the HMGB1 acetylation (Yu et al. 2019). RSV administration has decreased the levels of acetylation and expression of HMGB1 through the signaling pathway of SIRT1.

### Role of S100B in hippocampus

The higher expression of S100B was observed in cases of brain injury, cerebral ischemia, and many neurological diseases, including the AD (Nishiyama et al. 2002). The S100β is a calcium-binding protein, expressed at a larger quantity in the brain, mainly by the astrocytes (Reeves et al. 1994). The in vitro studies have shown that S100B maintains cell structure, growth, energy metabolism, and calcium homeostasis (Zimmer et al. 1995). It has been demonstrated that, in case of brain damage, S100B is passively released in large quantities, mainly from the astrocytes. The S100B participates in amplifying the inflammatory response by further activating the microglia and astrocytes (Steiner et al. 2011; Donato and Heizmann 2010). It was reported that, addition of S100B dimers to primary and established cultural cells has induced extension of the neurites. This in vitro activity was also repeated in the vivo at the hippocampus of the transgenic mice model (Reeves et al. 1994). It was reported that overexpression of S100B can cause neuronal damage and mimics the signs and symptoms of AD. The removal of S100B increases the synaptic plasticity and improves the learning and memory in the hippocampus. The long-term potentiation in the hippocampus was increased in the S100B removed mice model (Nishiyama et al. 2002).

### Effect of glucocorticoids and other hormones on hippocampus

The glucocorticoid hormones secreted by the adrenal cortex because of stress can dictate cognitive function. The hippocampus is highly vulnerable to chronic exposure to endogenous glucocorticoids (Libro et al. 2017). Elevated levels of basal endogenous glucocorticoids were observed in AD patients. This was correlated to the dysregulation of the hypothalamic–pituitary–adrenocortical pathway and the degeneration of the hippocampus and decreased cognitive and memory function. The pre-clinical studies have demonstrated that higher endogenous glucocorticoids can lead to amyloid-beta (Aβ) in the AD rat models by facilitating the amyloidogenic pathway. The clearance of Aβ through transcriptional mechanisms of glucocorticoid receptors is also decreased (Libro et al. 2017). It has been demonstrated that dexamethasone decreases the dendritic spine density and induces the proliferation and activation of microglia in the CA1 region in the triple-transgenic mouse model (3×Tg-AD) mice. Exposure to chronic stress and subsequent over-secretion of glucocorticoids severely impacts the synaptic structure, function, and plasticity in the hippocampus (Canet et al. 2018).

Hormones act as signalling factors for the neuroendocrine system, which modulate adult neurogenesis. There is a close relationship between the sex hormones and hippocampal neuronal proliferation in the hippocampus. It was reported that; increased level of ovarian hormones had increased the cell proliferation in the sub-granular region. Decreased levels of testosterone had reduced the capacity of new neuron production (Shohayeb et al. 2018). It is also demonstrated that mifepristone strongly enhances the dendritic spine density and improves the behavioral performance of 3×Tg-AD mice (Pedrazzoli et al. 2019). In the oligomeric amyloid-β peptide (oAβ25-35) rat model, mifepristone restored the basal levels of circulating corticosterone levels and repaired the apoptosis and synaptic deficits in the hippocampus (Canet et al. 2018).

### Proneurogenic lifestyle and neuronal degeneration

The lifestyle controls the human brain's function. Physical activity, food habits, and exposure to stress can impact the cognitive reserve of the brain. The animal model research has shown that running exercise can increase the hippocampal neurogenesis. The pro-neurogenic lifestyle will control the capability of the brain to prevent the neuronal degeneration due to the trauma, aging, and diseases (Valero et al. 2016). It was reported that, the low calorie diet and physical exercise can promote the adult neurogenesis. The omega-3 polyunsaturated fatty acids are required for the proper neuronal function as they exert an anti-inflammatory effect (Valero et al. 2016). It was reported that physical activity has prevented the decline of neurogenesis and boosted the memory in aged rodents and mouse AD models (van Praag 2008). Stress and severe exercise can elevate the glucocorticoids and affect the microglia (Gleeson et al. 2011). Both acute and chronic stresses decrease the adult neurogenesis due to the reduction in proliferation of neuroprogenitor cells and survival of newborn cells (Schoenfeld and Gould 2012). Animal research has shown that the high fatty diet has decreased the cell proliferation, cell survival and increased apoptosis in the DG (Park et al. 2011). It was observed that the high-fat diet decreased the BDNF and omega-3 polyunsaturated fatty acids increased it. This suggests that diet has a role in the maintenance of BDNF levels. Obesity and high-fat diet are associated with the state of chronic systemic inflammation, which increases the levels of pro-inflammatory cytokines like IL1-β and tumor necrosis factor α (TNFα) by the adipose tissue (Gregor and Hotamisligil 2011).

## Conclusion

The devastating neuronal loss and gliosis in the hippocampus are the hallmark of AD. The advanced shrinkage of the hippocampus is accountable for the short-term memory loss. The microglia in the brain phagocytose the Aβ aggregates and clears them from the neurons. But the microglia have dual nature as they help initially by phagocytosing the cellular debris in the brain and become harmful in the later stages of this degenerative disorder. Future studies, which throw insight into the microglial functions, will help understand AD’s management. The local and extrinsic environmental factors highly regulate hippocampal neurogenesis. Physical exercise, growth factors, and even hormones can influence neuronal production and survival in the DG, which controls the hippocampal-dependent cognitive and emotional functions.
